# First Molecular Detection of *Babesia ovis*, *Theileria* spp., *Anaplasma* spp., and *Ehrlichia ruminantium* in Goats from Western Uganda

**DOI:** 10.3390/pathogens9110895

**Published:** 2020-10-27

**Authors:** Maria Agnes Tumwebaze, Benedicto Byamukama, Dickson Stuart Tayebwa, Joseph Byaruhanga, Martin Kamilo Angwe, Eloiza May Galon, Mingming Liu, Seung-Hun Lee, Aaron Edmond Ringo, Paul Franck Adjou Moumouni, Jixu Li, Yongchang Li, Shengwei Ji, Patrick Vudriko, Xuenan Xuan

**Affiliations:** 1 National Research Center for Protozoan Diseases, Obihiro University of Agriculture and Veterinary Medicine, Obihiro, Hokkaido 080-8555, Japan; tumwebazeaggie@gmail.com (M.A.T.); benards.benedicto4@gmail.com (B.B.); eloizagalon@gmail.com (E.M.G.); lmm_2010@hotmail.com (M.L.); ggabheal@gmail.com (S.-H.L.); aringo2002@yahoo.com (A.E.R.); chakirou82@yahoo.fr (P.F.A.M.); JixuLi@hotmail.com (J.L.); yongchangli8762017@outlook.com (Y.L.); Jishengwei0903@hotmail.com (S.J.); 2 Research Center for Tropical Diseases and Vector Control, College of Veterinary Medicine, Animal Resources and Biosecurity, Makerere University, Kampala 7062, Uganda; tayebwa.dickson@gmail.com (D.S.T.); josephjbvincent@gmail.com (J.B.); mangwe@covab.mak.ac.ug (M.K.A.); 3 Department of Veterinary Pharmacy, Clinical & Comparative Medicine, School of Veterinary Medicine and Animal Resources, College of Veterinary Medicine, Animal Resources and Biosecurity, Makerere University, Kampala 7062, Uganda; 4 College of Veterinary Medicine, Chungbuk National University, Cheongju 28644, Korea; 5 Zanzibar Livestock Research Institute, Ministry of Agriculture, Natural Resources, Livestock and Fisheries, P.O. Box 159 Zanzibar, Tanzania

**Keywords:** *Babesia*, *Theileria*, *Anaplasma*, *Ehrlichia*, goats, Uganda

## Abstract

Ticks and tick-borne diseases are major impediments to livestock production. To date, there have been several studies on the prevalence of tick-borne pathogens (TBPs) in cattle, but very few studies have documented TBPs in goats in Uganda. In this study, polymerase chain reaction assays and sequence analysis of different molecular markers were used to assess the presence and genetic characteristics of TBPs in 201 goats from Kasese district in western Uganda. The risk factors associated with TBP infections were also analyzed. We detected *Theileria* spp. (13.4%), *Anaplasma phagocytophilum* (10.9%), *Anaplasma ovis* (5.5%), *Babesia ovis* (5.5%), and *Ehrlichia ruminantium* (0.5%). The sequences of *B. ovis* ssu rRNA and *A. ovis* *msp4* genes showed some degree of diversity among the parasite isolates in this study. The *E. ruminantium pCS20* sequence formed a well-supported clade with isolates from *Amblyomma variegatum* ticks from Uganda. Wildlife interaction, sampling location, low body condition score, tick infestation, and herd size were significantly associated with TBP infections in the goats. The findings in this study provide important information on the epidemiology of tick-borne pathogens in Uganda, and show that goats could be potential reservoirs for tick-borne pathogens.

## 1. Introduction

Ticks are one of the most important ectoparasites transmitting diseases of economic importance to livestock in Africa [1]. Of these, the most common tick-borne diseases (TBDs), considered as major impediments to livestock production and health in sub-Saharan Africa are babesiosis, theileriosis, anaplasmosis, and ehrlichiosis [2,3]. The losses caused by ticks and TBDs are largely due to high mortality, reduced production, cost of treating the sick animals, and control of vectors [4].

Babesiosis in small ruminants is caused by several *Babesia* species: *Babesia ovis*, *B. motasi*, and *B. crassa* [5]. *B. ovis* is highly pathogenic, especially in sheep, and is principally transmitted by *Rhipicephalus bursa*; clinical manifestation of the disease is characterized by fever, anemia, icterus, and hemoglobinuria, with mortalities ranging from 30% to 50% in the field [6,7]. *B. motasi* causes acute to chronic disease, whereas *B. crassa* has low pathogenicity [8].

*Theileria* species known to infect small ruminants include *Theileria lestoquardi, T. ovis, T. luwenshuni, T. uilenbergi, Theileria* sp. OT3, *Theileria* sp. MK, and *T. separata* [9,10,11]. *Theileria lestoquardi, T. luwenshuni*, and *T. uilenbergi* have been reported as pathogenic. *Theileria lestoquardi* is the most pathogenic, which is mainly attributed to the occurrence of malignant theileriosis in sheep [8]. *Theileria* ovis is the main and most common etiological agent of ovine theileriosis, causing considerable economic losses among sheep and goats in tropical and sub-tropical areas [12]. Although not as pathogenic as *T. lestoquardi*, the disease caused by *T. ovis* manifests with fever, progressive weight loss, and decrease in production, and may lead to death.

Anaplasmosis in small ruminants is caused by *Anaplasma ovis*, an intraerythrocytic rickettsial pathogen considered to be the most frequent cause of small ruminant anaplasmosis [13]. The acute phase of the disease is characterized by anemia, fever, weight loss, abortion, low milk production, jaundice, and sometimes, death [14]. The pathogen has various transmission routes: ticks, biting insects, blood inoculation, or exposure to blood-contaminated fomites. Animals that recover from the disease usually remain carriers [8,15]. *Anaplasma phagocytophilum* is an obligate, intracellular, Gram-negative bacterium that causes tick-borne fever in domestic and wild ruminants. It also causes human, canine, and equine granulocytic anaplasmosis. The disease is characterized by high fever, dullness, anorexia, and reduced milk production in domestic animals [16].

Ehrlichiosis is an important disease, caused by *Ehrlichia ruminantium*, that normally starts with a sudden onset of clinical signs, followed by death if the animal is not attended to. Ehrlichiosis affects domestic and wild ruminants in sub-Saharan Africa, as well as some Indian and Caribbean islands [17].

In Uganda, more than 30% of calf crop loss in cattle is due to TBDs, and almost 90% of the total disease control costs are due to the same diseases [4]. Unfortunately, this data is limited to cattle. Previous studies in the country have mostly focused on TBD research in bovines, while small ruminants have received limited attention. Small ruminants, especially goats (*Capra aegagrus hircus*), play a very important role in poverty alleviation, nutrition, and animal production in Uganda [18], but research into diseases affecting their health is neglected. Approximately 90% of the national livestock herd is kept under pastoral and mixed smallholder farms [19], but these kinds of systems increase the risk of inter-species disease transmission. This is attributed to some animals having a carrier status, and thereby serving as reservoirs of pathogens and sources of infection for the naïve ones. Therefore, this study investigated the tick-borne pathogens (TBPs) infecting goats, the genetic characteristics of the isolated pathogens, and risk factors associated with the infections.

## 2. Results

### 2.1. Sampled Animal and Farm Demographics

**Figure 1:**
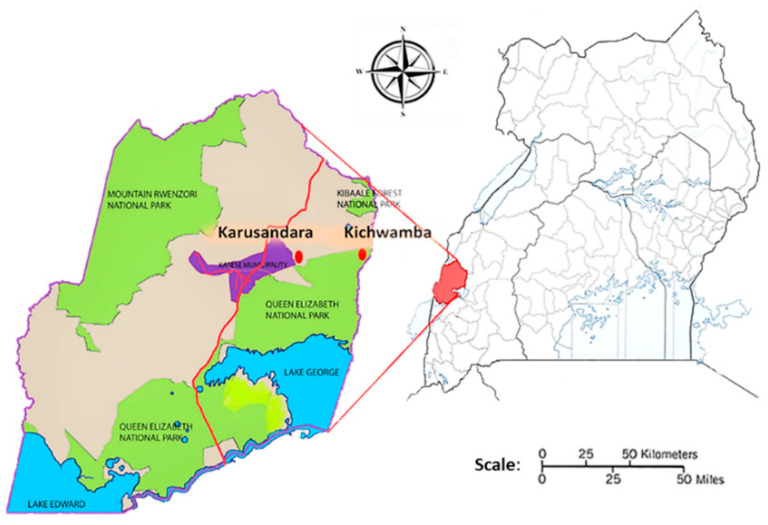
A map of Uganda showing the two subcounties in the Kasese district where samples were obtained.

Throughout the study, 201 goats were sampled from Karusandara and Kichwamba subcounties (Figure 1). Among the goats sampled, 51.2% (103/201) were from Karusandara subcounty and 48.8% (98/201) were from Kichwamba subcounty. Local breeds accounted for 80.6% (162/201) and crossbreeds for 19.4% (39/201) of the goats sampled. Meanwhile, 90.5% (182/201) of the goats were female and 9.5% (19/201) were male. Young goats below one year old accounted for 55.7% (112/201) of the sampled animals, and those above one year were 44.3% (89/201). From our assessment, 79.6% (160/201) of the animals had a body condition score (BCS) equal to or above three (three indicating optimal BCS) and 20.4% (41/201) had a score below three. On the other hand, 38.8% (78/201) were infested with ticks (Table 1), while of the 19 farms sampled, 63.2% (12/19) applied acaricides on goats for controlling ticks (Table 2).

The majority of the farms were mixed smallholder farms, with 73.7% (14/19) having a herd size of ≥30 while 26.3% (5/19) had a herd size of <30 goats. Free-range grazing (52.6%, 10/19) was the most practiced grazing system, followed by paddocking (21.1%, 4/19), communal grazing (15.8%, 3/19), and tethering (10.5%, 2/19). Farmers had a perception that goats did not easily succumb to diseases. The survey also showed that 57.9% (11/19) of the farms did not have any disease challenges. However, eight farms had disease challenges; malaise, which the farmers referred to as fever, accounted for 50.0% (4/8). This was followed by helminthiasis at 25.0% (2/8), then abortion and abscesses, each at 12.5% (1/8). Within the areas sampled, 47.6% (9/19) of the farms had their animals interact with wildlife, and 52.6% (10/19) had none or did not know of any interaction with wildlife. Among the wildlife encountered, antelopes and elephants (each at 33.3%, 3/9) were the most frequent, followed by warthogs, buffaloes, and hares, each at 22.2% (2/9) (Table 2).

### 2.2. Molecular Detection of Tick-borne Pathogens (TBPs) in Goats

Overall, polymerase chain reaction (PCR) analysis revealed that 30.4% (61/201) of the goats were infected with at least one pathogen. *Theileria* spp. were the most prevalent pathogen, at 13.4% (27/201), followed by *A. phagocytophilum* at 10.9% (22/201), *B. ovis* at 5.5% (11/201), *A. ovis* at 5.5% (11/201), and *E. ruminantium* at 0.5% (1/201) (Table 3). All 27 samples that were positive for *Theileria* spp. tested negative for *T. ovis* ssu rRNA and *T. lestoquardi* 18S rRNA. However, six dual infections and a single triple infection were detected (Table 4). Notably, goats from farms in Karusandara had significantly higher infection rates (*p* = 0.00001) than those from Kichwamba (Table 3).

### 2.3. Sequence Similarities and Phylogenetic Analysis

Four to seven isolates were used as representatives of the different detected pathogens for sequence analysis. The four partial sequences of the ssu rRNA gene fragment of *B. ovis* isolates shared 96.5–99.1% similarity among themselves and 97–100% similarity to isolates from Tunisia (KF723611), Turkey (MG569902 and AY9998123), and Iran (KY581552). Those of the *msp4* gene of the six *A. ovis* isolates shared 89.9–100.0% similarity among themselves, and 99.71% similarity to isolates from sheep in Mongolia (LC412082) and goats from Sudan (KU497708). Seven *Theileria* spp. isolates were subjected to sequence analysis, wherein four (MT239524–MT239527) showed 99.3–99.5% similarity to *T. mutans,* one (MT239528) had 99–100% similarity to *T. annulata* isolates from different countries, another (MT239529) showed 98.3% similarity to *Theileria* sp. Sola and 97.3% to *T. capreoli* isolates, and finally, one (MT239530) showed 99.1–99.3% similarity to *T. parva* vaccine strains from East Africa. The *E. ruminantium pCS20* isolate had 99% similarity to isolates from several mammalian hosts from different African countries, tick vectors from Uganda (MK371033 and MK371028), a Sankat strain from South Africa (AY236065), and a goat isolate from Sudan (MG383909).

**Figure 2:**
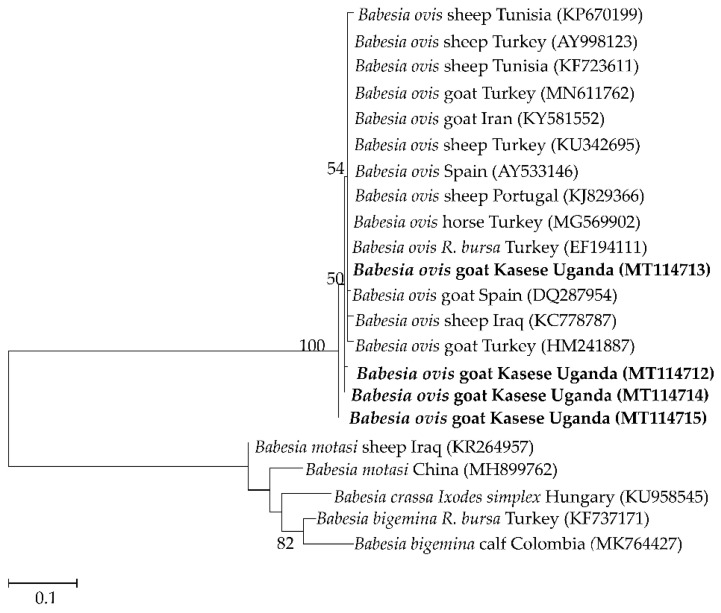
Phylogenetic analysis of *Babesia ovis* based on the ssu rRNA. The tree was constructed with maximum likelihood with the Tamura method using Mega X (Penn State University, PA, USA). The sequences obtained in this study are shown in bold; number at the nodes represent the occurrence of clades at 1000 bootstrap replications.

**Figure 3:**
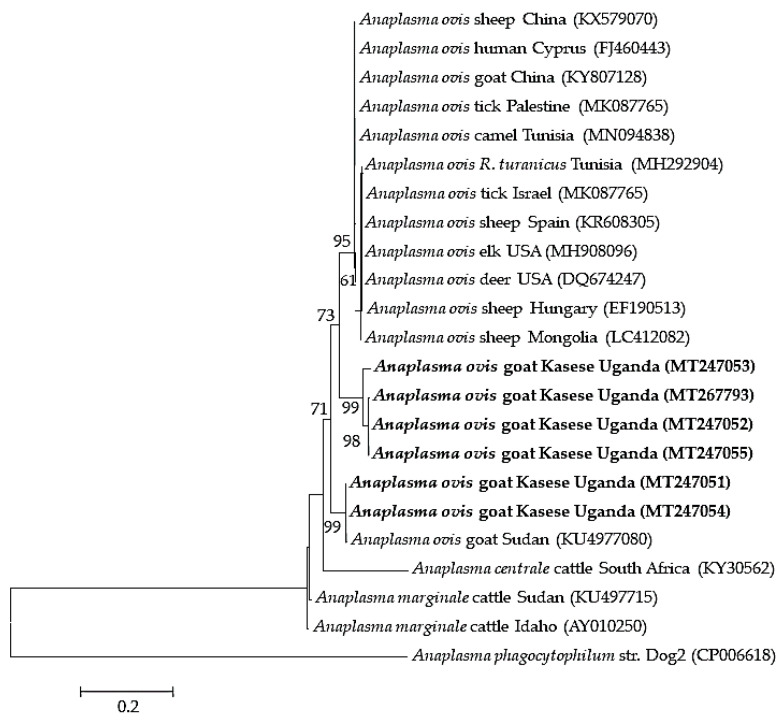
Phylogenetic analysis of *Anaplasma ovi*s based on the *msp4* gene. The tree was constructed with maximum likelihood with the Kimura-2 method using Mega X (Penn. State University). The sequences obtained in this study are shown in bold; number at the nodes represent the occurrence of clades at 1000 bootstrap replications.

**Figure 4:**
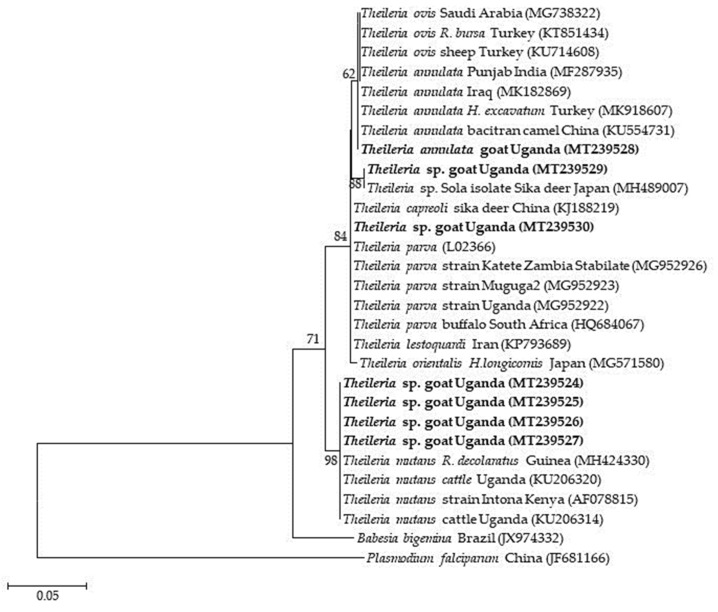
Phylogenetic analysis of *Theileria* spp. based on the 18S rRNA gene. The tree was constructed with maximum likelihood with the Tamura-Nei method, using Mega X (Penn. State, University). The sequences obtained in this study are shown in bold; number at the nodes represent the occurrence of clades at 1000 bootstrap replications.

Phylogenetic analysis revealed that the *B. ovis* isolates (MT114712–MT114715) clustered together with *B. ovis* isolates from Turkey and the first isolate of *B. ovis* in Africa, isolated from sheep in Tunisia (KF723611) (Figure 2). The *A. ovis msp4* sequences formed two separate clades. Four of the sequences clustered together, and the other two clustered with a goat isolate from Sudan (Figure 3). Meanwhile, four of the *Theileria* spp. isolates (MT239524–MT239527) clustered together with *T. mutans* isolates from Kenya, Guinea, and Uganda, while MT239528 clustered together with *T. annulata* sequences. MT239529 also clustered with a *Theileria* sp. Sola isolate from a sika deer in Japan. On the other hand, MT239530 clustered together with *T. parva* vaccine strains and a buffalo isolate from South Africa (Figure 4).

**Figure 5:**
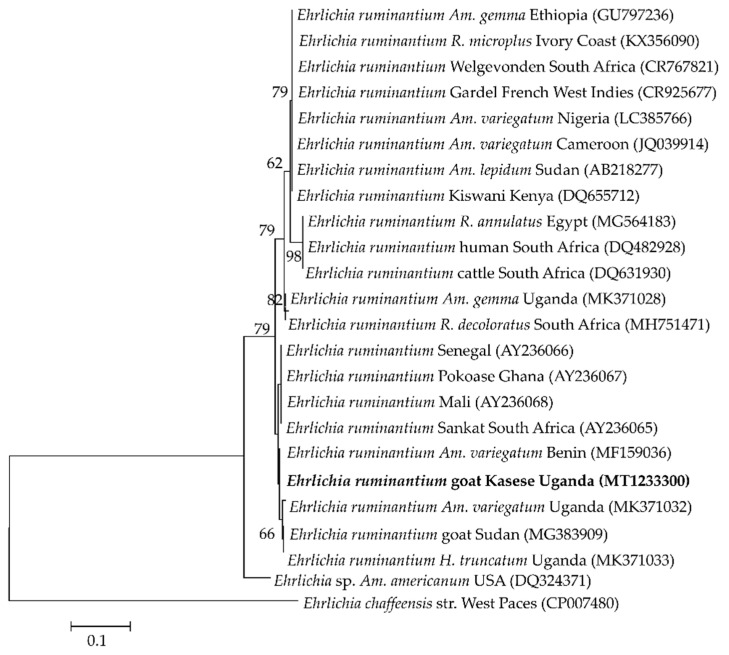
Phylogenetic analysis of *Ehrlichia ruminantium* based on the *pCS20* gene. The tree was constructed with maximum likelihood with the Kimura-2 method using Mega X (Penn. State, University). The sequence obtained in this study is shown in bold; number at the nodes represent the occurrence of clades at 1000 bootstrap replications.

The *E. ruminantium* isolate from this study was closely related to isolates from different vectors from Uganda and other African countries (Figure 5).

### 2.4. Risk Factors Associated with the Detected TBPs

Multivariate logistic regression analysis revealed that the rate of *Theileria* infection was significantly higher in goats from Karusandara (*p* = 0.003). These goats were 20 times more likely to have *Theileria* spp. infection compared to those from Kichwamba (Table 5). Meanwhile, *B. ovis* and *A. phagocytophilum* infections were significantly associated with wildlife interaction. Goats that interacted with wildlife were ten and eight times more likely to be infected with *B. ovis* and *A. phagocytophilum*, respectively. Animals with low body condition score (BCS) (<3) were also four times more likely to have *Theileria* spp. infection. Notably, even though the breed, tick infestation, and herd size were significantly associated with *A. phagocytophilum* infection, the odds ratio for occurrence of the infection were relatively low. There was a similar observation for the relationship between *Theileria* spp. infection and tick infestation (Table 5). Conversely, there was no significant relationship between *A. ovis* and *E. ruminantium* infection with any of the analyzed risk factors in this study (Table 5).

## 3. Discussion

Tick-borne pathogens can infect both domestic and wild animals, with worldwide geographical distributions mirroring that of their tick vectors. In sub-Saharan Africa, tick-borne diseases are major constraints to livestock production, including goats [1]. One of the biggest challenges to controlling these devastating diseases in this region is the limited information on their epidemiology. In the present study, we report the molecular detection and genetic characteristics of *B. ovis*, *A. phagocytophilum*, *Theileria* spp., *A. ovis*, and *E. ruminantium* infecting goats from the Kasese district, Uganda.

*Babesia ovis* is highly pathogenic, causing significant losses in small ruminants [20,21]. In the current study, we detected *B. ovis* in 5.5% of the goats. To the best of the authors’ knowledge, this is the first report of *B. ovis* detection in sub-Saharan Africa. In Africa, *B. ovis* was first detected in goat samples from Tunisia, with a detection rate (7.8%), slightly higher than the 5.5% *B. ovis* detection rate in the current study [22]. The pathogen was recently detected in *Rhipicephalus bursa* and *R. turanicus* ticks from Algeria [23], tick species that have been identified as important vectors of *B. ovis* [22,24]. In addition to North Africa, *B. ovis* was previously reported in the Mediterranean region and Middle East [25,26,27]. The epidemiology of *B. ovis* is reported to be closely related to the ecology of its tick vector, mainly during the active period (April to July) of the adult tick, *R. bursa* [28]. The same report emphasized that the disease mainly occurs in infested areas. *R. bursa* tick population and distribution is influenced by specific vegetation and climatic conditions [6], explaining the limited distribution of *R. bursa* to the Mediterranean region. The current detection of *B. ovis* in goats from Kasese, Uganda suggests possible presence of other tick vectors, *R. turanicus* and *R.* *sanguineus*, in this district. Nevertheless, basing on the argument from the same study that “the epidemiology of ovine babesiosis due to *B. ovis* is closely related to the bio-ecology of *R. bursa”* [6], we cannot overlook the possibility of evolvement of new *R. bursa* strains suitable for such climatic conditions. On the other hand, phylogenetic analysis showed that one of the *B. ovis* isolates, MT114713, is closely related to an isolate from *R. bursa* in Turkey (EF194111), while MT114712 and MT114714 showed a closer relationship to an isolate from a goat in Turkey (HM241887). MT114715 formed a different subclade (Figure 2). The isolates showed some degree of diversity; however, the mode of transmission of the pathogen remains unclear. Whether it as an introduction of a strain from goats imported with the infection or with infected *R. bursa* ticks, or even the possibility of transmission from the wild, remains to be investigated. Further studies are required to identify specific vectors, strain diversity, and distribution of this pathogen, not only in goats but also in sheep in Uganda.

*Anaplasma phagocytophilum* can cause up to 30% lamb mortality in sheep [29]. In Africa, *A. phagocytophilum* has been reported in dogs, horse, cattle, and ticks from the northern part of the continent [30,31]. This study recorded a higher detection rate (10.9%) in goats than these reports. Conversely, a higher detection rate was reported in goats from northern China [32]. *Anaplasma phagocytophilum* is primarily transmitted by *Ixodes* ticks, which have not been reported in Uganda. Notably, *Ixodes* ticks are not the only vectors, as *Anaplasma* bacteria can be mechanically transmitted by biting flies and potentially by other tick species [30,33]. In light of the current observation, studies are required to assess the vector competence of the potential tick species for *A. phagocytophilum* in Uganda. Importantly, there is also a need to assess the potential risk of transmission of this pathogen to humans.

Statistical analysis further revealed that goats that interacted with wildlife were more likely to be infected with *B. ovis* and *A. phagocytophilum* (Table 5). Wildlife has been implicated as a possible reservoir for TBPs [34], although to date, studies to confirm this observation have yet to be conducted in Uganda. *Anaplasma phagocytophilum* was previously isolated from wild animals in Europe [35]. Therefore, there is a need to investigate and elucidate the relationship between transmission and infection of *B. ovis* and *A. phagocytophilum* strains at the wildlife–livestock interface in Uganda. Tick infestation has been associated with the occurrence of anaplasmosis [36]. This agrees with our finding that *A. phagocytophilum* infection was significantly higher in goats that were infested with ticks as compared to those that were not. Farms with more than thirty goats were more likely to have animals infected with *A. phagocytophilum*. Although the odds and confidence intervals were low, our study agrees with a report [37] that observed that there is crowding of animals in large herds. This increases the risk of transmission of parasites between the infected and non-infected animals. Therefore, farmers, especially those with big herd sizes, require sensitization on proper farm management as an effort towards control of tick-borne diseases.

Theileriosis is an economic concern in parts of the world where the livelihood of an economically disadvantaged population is largely dependent on animal products [38]. The overall detection of *Theileria* spp. in this study was 13.4%, which is higher than the previously detected 2% in goats in Ethiopia [39]. A previous study from Uganda reported comparable result (10.0%) in goats [40]. In contrast, higher infection rates (58–71.4%) have been reported in sheep from neighboring Kenya [41]. Sequence analysis revealed the presence of *T. annulata*, *Theileria* spp. strains closely related to *T. parva* vaccines strains, *T. mutans*, and *Theileria* sp. Sola-related species in the goats sampled in the present study. A study from Iran showed that *T. annulata* could successfully transform goat peripheral blood mononuclear cells and cause infection [42], although it is known to infect bovines. The results from that study could explain the presence of the pathogen in goats in the present study. Our finding also corroborates the detection of *T. annulata* in sheep in Iran and Sudan [9,43]. As *T. parva* vaccines are only administered to cattle in Uganda, obtaining an isolate closely related to the vaccine strains in the sampled goats suggests the possibility of cross-species transmission of *T. parva*. This can be attributed to goats and cattle commonly sharing grazing grounds and *Rhipicephalus appendiculatus* tick vector in Uganda. However, whether *T. parva* can successfully transform lymphocytes and cause clinical infection in goats still remains to be studied. *T. mutans* is widespread in Africa, and only causes benign theileriosis in cattle, but the pathogen was previously identified in sheep [44]. Unfortunately, there have not been many studies to confirm this finding. We also identified an isolate closely related to *Theileria* sp. Sola, which has been reported in a sika deer from Japan [45], suggesting that wildlife could be infected with or serve as reservoirs for this pathogen. The establishment of *T. mutans* and *Theileria* sp. Sola in goats and the vectors responsible for transmission, especially for *Theileria* sp. Sola-related species, require further investigation. *T. ovis*, the most common cause of theileriosis in small ruminants in countries like Sudan [46], and *T. lestoquardi*, the most virulent species, especially in sheep [47], were not identified in this study. To the best of our knowledge, *T. lestoquardi* has only been detected in North Africa [48,49]. On the contrary, *T. ovis* has been detected in cattle in neighboring Tanzania [50], and in small ruminants in North Africa and northern Ethiopia [39,43,51,52]. The non-detection of these two *Theileria* species in the positive samples suggests the possibility of absence or very low prevalence levels of the pathogens in goats in this area.

Goats from Karusandara were 20 times more likely to succumb to theileriosis than those from Kichwamba. This finding can be attributed to farm management practices, as most farmers in Karusandara use free-range grazing (Table 2), which increases the exposure of animals to ectoparasites and consequently, to risk of infection [53]. Animals with body condition scores below three were four times more likely to suffer from theileriosis. A previous study showed that low body condition score was associated with the prevalence of ectoparasites, including ticks, which increases the risk of pathogen transmission to the animals [54].

*Anaplasma ovis* is the major cause of small ruminant anaplasmosis [11]. *Anaplasma ovis* detection in this study (5.5%) was lower than previously reported detection rates in goats from South Africa (36.3%) [55], Sudan (60.1%) [51], and Tunisia (93.8%) [56]. The differences in infection rates could be attributed to the difference in climatic conditions, vector distribution, and sample size. Continuous surveillance is required to give a clearer picture of the prevalence of *A. ovis* in small ruminants in the country. The phylogenetic analysis of the *msp4* gene of the *A. ovis* isolates revealed some degree of diversity among *A. ovis*, showing two different genotypes infecting goats in Kasese (Figure 3). The *msp4* gene is routinely used to genetically characterize *Anaplasma* spp. [57]. Several studies have shown a similar trend in *A. ovis msp4* genotypes infecting small ruminants; in Tunisia, two genotypes have been identified [31]. Another study in China also identified two genotypes of *A. ovis msp4* isolates, where the authors attributed the low genetic diversity of the *msp4* gene to low prevalence of *A. ovis* [58], which could explain the findings in our study. This observation corroborates our findings, suggesting the possibility of genotypic variation among *A. ovis* genotypes or strains infecting goats in Kasese. Therefore, using more genetically diverse genes like *msp1a* would be crucial in elucidating the diversity and epidemiology of *Anaplasma* infections in small ruminants in Uganda.

In this study, only one goat from Kichwamba subcounty was positive for *E. ruminantium*. This finding is similar to a previous report from West Kordofan, Sudan [51]. *Ehrlichia ruminantium* is transmitted by *Amblyomma* ticks, of which the most widespread species is *Amblyomma variegatum* [17]. Notably, *Am. Variegatum* has been identified in Western Uganda [59]. More importantly, phylogenetic analysis of the *pCS20* gene revealed that the isolate in this study was closely related to *E. ruminantium* isolated from *Am. Variegatum* ticks from Uganda, which suggests possible transmission by this tick species. However, the fact that only one goat was infected requires further investigation to elucidate the distribution of *E. ruminantium* in small ruminants in Uganda, as this is the first report of molecular detection of *E. ruminantium* from goats in the country.

This study reports the first molecular detection and characterization of several TBPs in goats in Uganda. We identified *Theileria* spp., *A. phagocytophilum*, *B. ovis*, *A. ovis*, and *E. ruminantium*. The findings of this study demonstrate that wildlife interaction, herd size, tick infestation, and body condition score are risk factors that play important roles in TBP infection in goats in Uganda. The current findings show that goats may play a vital role as reservoirs of tick-borne pathogens.

## 4. Materials and Methods

### 4.1. Ethical Statements

In this study, the owners of the selected farms were informed about the study and gave their consent to sampling their animals. Approval was obtained from theMinistry of Agriculture, Animal Industry and Fisheries (MAAIF) Uganda (IHVC-Products No. 00044840 on 05/06/2019 to carryout research on the samples collected in Japan. All laboratory procedures were carried out according to the ethical guidelines on the use of animal samples of Obihiro University of Agriculture and Veterinary Medicine (permit for the animal experiment: 19-15, DNA experiment: 1724-3).

### 4.2. Sampling Area and Study Design

A cross-sectional study was conducted in Kasese district in Western Uganda, from May to June 2019. To obtain the desired samples, our team visited farmers in Karusandara and Kichwamba subcounties. Karusandara subcounty is 6 km away from Queen Elizabeth National Park (QENP), and Kichwamba is located within the vicinity of the National Park (Figure 1). Kasese district has a tropical climate, good for vegetation and therefore, favorable for livestock, wildlife, and several microorganisms’ survival.

Two parishes were randomly selected by the District Veterinary Officer (DVO) from two subcounties, Karusandara and Kichwamba: Karusandara and Kibuga parishes for Karusandara subcounty, and Ibuga and Hima parishes for Kichwamba subcounty. Nineteen farms were randomly selected from the parishes, and the number of animals sampled varied depending on the herd size and availability of the animals in the respective farms at sampling time. Animals were randomly chosen, restrained, and examined for parameters like age, body condition score, breed, sex, and tick infestation. The majority of the animals sampled were clinically healthy.

### 4.3. Sample Collection and DNA Extraction

Open-ended questionnaires were used to briefly interview the farmers, with their consent, on the farming system, tick control methods (if any), disease challenges commonly faced, wildlife interaction, common species that interact with the goats, and diseases encountered at the farm. Animal profiles were also recorded: age, sex, tick infestation, breed, and body condition score (BCS). Scoring of the BCS was done using a scale of 1 to 5 (BCS 1 = extremely thin goat with no fat reserves, BCS 3 = health looking goat, and BCS 5 = excessively fat/obese goat). Whole blood samples (*n* = 201) were collected into EDTA (Ethylenediaminetetraacetic acid) tubes from the jugular vein of goats. These were kept in a cool box and transported to the Research Center for Ticks and Tick-Borne Diseases, Makerere University, Uganda, for DNA extraction. DNA was extracted from 200 µL of the blood using the QIAamp DNA Blood Mini Kit (Qiagen, Hilden, Germany), following the manufacturer’s protocol. The DNA was transported to the National Research Center for Protozoan Diseases (NRCPD), Obihiro University of Agriculture and Veterinary Medicine, Japan, and stored at −30 °C until use.

### 4.4. Polymerase Chain Reaction Assays and DNA Sequencing

PCR assays were done using species- or genus-specific primers targeting *B. ovis, Theileria* spp., *T. ovis*, *T. lestoquardi*, *A. ovis, A. phagocytophilum*, and *E. ruminantium*. The gene targets, amplicon sizes, and references are shown in Table 6.

The PCR reaction mixture prepared was composed of 0.5 U of *Taq* polymerase (New England BioLabs, Ipswich, MA, USA), 10 µM of each of the forward and reverse primers, 200 µM deoxyribonucleotide triphosphates (dNTPs), 10× ThermoPol Buffer (New England BioLabs, Ipswich, MA, USA), a DNA sample, and double-distilled water up to 10 µL. Double-distilled water was used as negative control. Positive isolates from previous studies—*T. ovis* (goat), *E. ruminantium* (goat) [51], *A. ovis* (goat) [55], and *B. ovis* (goat) [60]—and previous lab isolates for *Theileria* spp. and *T. lestoquardi*, were used as positive controls. PCR amplicons were run in 1.5% agarose gel electrophoresis and stained in ethidium bromide, after which they were visualized under UV light.

Positive amplicons were extracted from agarose gel using QIAquick Gel Extraction Kit (Qiagen). The concentration of the extracts was measured using the NanoDrop 2000 spectrophotometer (Thermo Fisher Scientific, Waltham, MA, USA). Sequencing PCR of the extracts was performed using BigDye Terminator Cycle Sequencing Kit (Applied Biosystems, Waltham, MA, USA). Nucleotide sequences were directly sequenced using the 3100xl Genetic Analyzer (Applied Biosystems) and aligned using MUSCLE in MEGA Version X software (Penn. State, University) [66].

Sequences identified in this study were deposited in the GenBank database of the National Center for Biotechnology Information, using BankIt. The GenBank accession numbers were assigned as follows: *B. ovis* ssu rRNA (MT114712-MT114715)*, Theileria* spp. 18S rRNA (MT239524-MT239530), *A. ovis msp4* (MT247051-MT247055 and MT267793), and *E. ruminantium* pCS20 (MT123300).

### 4.5. Phylogenetic Analysis

The phylogenetic trees were constructed based on the maximum likelihood method (Kimura-2 parameter and Tamura-Nei models), using the MEGA Version X program; sequences were aligned using Muscle program, and the evolutionary models were selected according to the best fit model, with the lowest BIC (Bayesian Information Criterion) scores and lowest gamma distribution value. Numbers on internal branches represent bootstrap values (only > 60% are shown), obtained at 1000 replications. The inclusion of the sequences for analysis was based on the host, region, and country. An appropriate outgroup was included.

### 4.6. Statistical Analysis

Univariate and multivariate logistic regression analysis using R statistical software was used to analyze the infection rates of the pathogens detected and associated risk factors. A *p*-value of <0.05 was considered as statistically significant.

## Figures and Tables

**Table 1:** Animal demographics: animal risk factors analyzed in the study.

Parameter	Number (*n* = 201)	Percentage (%)
**Source**		
**Karusandara**	103	51.2
**Kichwamba**	98	48.8
**Breed**		
**Local**	162	80.6
**Cross**	39	19.4
**Sex**		
**Female**	182	90.5
**Male**	19	9.5
**Age**		
**Young**	112	55.7
**Adult**	89	44.3
**Body condition score (BCS)**		
**≤3**	160	79.6
**>3**	41	20.4
**Tick infestation**		
**Infested**	78	38.8
**Not infested**	123	61.2

Key: BCS score index (1 = extremely thin goat, 3 = healthy looking goat, 5 = excessively fat/obese goat).

**Table 2:** Farm demographics: results of the brief farm survey on farming systems, wildlife interaction, tick control, and disease challenges.

Parameter	No. of Farms	Percentage (%)
**Farming system**	(*n* = 19)	
**Free range**	10	52.6
**Paddocking**	4	21.1
**Communal**	3	15.8
**Tethering**	2	10.5
**Herd size**		
**≥30**	14	73.7
**<30**	5	26.3
**Acaricide application**		
**Application**	12	63.2
**None**	7	36.8
**Interaction with wildlife**		
**Interaction**	9	47.4
**None**	10	52.6
**Disease challenges**		
**Observed**	8	42.1
**None**	11	57.9
**Wildlife Encountered**	(*n* = 9)	
**Antelopes**	3	33.3
**Elephants**	3	33.3
**Warthogs**	2	22.2
**Buffaloes**	2	22.2
**Hares**	2	22.2
**Primary Disease challenges**	(*n* = 8)	
**Fever**	4	50.0
**Helminths**	2	25.0
**Abortion**	1	12.5
**Abscesses**	1	12.5

**Table 3:** Detection of tick-borne pathogens based on sampling locations.

	Overall (%)	*Babesia ovis* (%)	*Theileria* spp. (%)	*Anaplasma ovis* (%)	*Anaplasma phagocytophilum* (%)	*Ehrlichia ruminantium* (%)
**Karusandara (*n* = 103)**	52 (50.5)	4 (3.9)	25 (24.3)	8 (7.8)	15 (4.9)	0 (0.0)
**Kichwamba (*n* = 98)**	20 (20.4)	7 (7.1)	2 (2.0)	3 (3.1)	7 (7.1)	1 (1.0)
**Total (*n* = 201)**	72 (35.8)	11 (5.5)	27 (13.4)	11 (5.5)	22 (10.9)	1 (0.5)
***p*-value**	0.00001 *	0.30985	0.00001 *	0.14260	0.09213	N/A ^1^

^*^p-value < 0.05 was considered statistically significant; ^1^ not analyzed.

**Table 4:** Types of tick-borne infections detected in goats.

Pathogen Species	No. of Positives (*n* = 201)	Detection Rate %
Single infections		
*Babesia ovis*	7	3.5
*Theileria* spp.	17	8.5
*Anaplasma ovis*	3	1.5
*Anaplasma phagocytophilum*	16	7.9
*Ehrlichia ruminantium*	1	0.49
Coinfections		
Double		
*Babesia ovis* + *Anaplasma ovis*	1	0.49
*Babesia ovis* + *Theileria* spp.	1	0.49
*Babesia ovis* + *Anaplasma phagocytophilum*	3	1.49
*Anaplasma ovis* + *Theileria* spp.	4	1.99
*Anaplasma ovis* + *Anaplasma phagocytophilum*	2	0.99
*Theileria* spp. + *Anaplasma phagocytophilum*	5	2.49
Triple		
*Theileria* spp. + *Anaplasma ovis* + *Anaplasma phagocytophilum*	1	0.49

**Table 5:** Multivariate logistic regression risk factor analysis for the tick-borne pathogen infections.

Pathogen	Parameter	*p*-Value	OR	Confidence Interval
*B. ovis*	Wildlife interaction	0.020	10.0	1.25–76.99
*Theileria* spp.	Subcounty	0.003	19.9	3.84–103.46
	BCS	0.011	3.90	1.35–11.42
	Tick infestation	0.04	0.22	0.05–0.94
*A. phagocytophilum*	Breed	0.005	0.18	0.05–0.60
	Tick infestation	0.030	0.30	0.09–0.93
	Herd size	0.023	0.16	0.03–0.78
	Wildlife interaction	0.001	7.97	2.15–29.54

*p*-value < 0.05 was considered statistically significant; OR: odds ratio.

**Table 6:** Target genes and primers used in the study.

Pathogen	Target Gene	Primer Sequence (5′->3′)	Annealing Temp (°C)	Amplicon Size (bp)	Reference
*Babesia ovis*	ssu rRNA	TGGGCAGGACCTTGGTTCTTCT CCGCGTAGCGCCGGCTAAATA	62	549	[61]
*Theileria* spp.	18S rRNA	GAAACGGCTACCACATCT AGTTTCCCCGTGTTGAGT TTAAACCTCTTCCAGAGT TCAGCCTTGCGACCATAC	55	778 581	[62]
*Theileria ovis*	ssu rRNA	TCGAGACCTTCGGGT TCCGGACATTGTAAAACAAA	53	520	[52]
*Theileria lestoquardi*	18S rRNA	GTGCCGCAAGTGAGTCA GGACTGATGAGAAGACGATGAG	52	730	[43]
*Anaplasma ovis*	*msp4*	TGAAGGGAGCGGGGTCATGGG GAGTAATTGCAGCCAGGCACTCT	62	347	[63]
*Anaplasma phagocytophilum*	16S rRNA	TCCTGGCTCAGAACGAACGCTGGCGGC AGTCACTGACCCAACCTTAAATGGCTG GTCGAACGGATTATTCTTTATAGCTTGC CCCTTCCGTTAAGAAGGATCTAATCTCC	50	1433 926	[64]
*Ehrlichia ruminantium*	*pCS20*	ACTAGTAGAAATTGCACAATCYAT RCTDGCWGCTTTYTGTTCAGCTAK ACTAGTAGAAATTGCACAATCYAT TGATAACTTGGWGCRRGDARTCCTT	61	400 278	[65]
